# Clinical evaluation of ankle arthrodesis with Ilizarov fixation and internal fixation

**DOI:** 10.1186/s12891-019-2524-1

**Published:** 2019-04-11

**Authors:** Piotr Morasiewicz, Maciej Dejnek, Wiktor Orzechowski, Wiktor Urbański, Mirosław Kulej, Szymon Łukasz Dragan, Szymon Feliks Dragan, Łukasz Pawik

**Affiliations:** 1 Department and Clinic of Orthopaedic and Traumatologic Surgery, Wrocław Medical University, ul. Borowska 213, 50-556 Wrocław, Poland; 2 Department of Physiotherapy and Occupational Therapy in Motor Disorders and Dysfunctions, University of Physical Education, Al. IJ Paderewskiego 35, Wroclaw, Poland

**Keywords:** Clinical, Ankle arthrodesis, Ilizarov fixation, Internal fixation

## Abstract

**Background:**

Ankle arthrodesis may have internal or external stabilization.

We assessed whether the type of stabilization after ankle arthrodesis will affect: (1) functional outcome in Foot and Ankle Ability Measure (FAAM) scale, (2) pain level, (3) period of hospitalization, (4) rate of complications.

**Methods:**

We retrospectively studied 47 individuals after ankle arthrodesis with Ilizarov fixation (group 1, *n* = 21) and internal stabilization (group 2, *n* = 26) at our institution in years 2007–2015. Clinical outcomes were measure by: (1) functional outcome in FAAM scale, (2) pain level, (3) period of hospitalization, (4) rate of complications.

**Results:**

Total number of complications in Ilizarov group was 13, which corresponded to 0.62 complications per patient on average. In group 2 there were 15 complications, which corresponded to 0.58 complications per patient on average. The intergroup difference in rate of complications was not statistically significant (*p* = 0.066). In group 1 the mean VAS pain level before treatment was 4.69 and after treatment was 1.5 (*p* = 0.037). In group with internal stabilization the mean VAS pain level before treatment was 4.71 and after treatment was 2.9 (*p* = 0.044). In group 1 the mean period of hospitalization was 5.29 days, in group 2 was 5.71 days (*p* = 0.517). In group 1 the mean functional outcome in FAAM scale was 79.38, in group 2 was 70.11 (*p* = 0.458).

**Conclusions:**

Ankle arthrodesis with Ilizarov stabilization is associated with lower prevalence of VAS pain level after surgery than after internal screws stabilization. Rate of complications, FAAM functional score and period of hospitalization were not statistically significant between group 1 and 2. Clinical outcome was satisfactory in group 1 and 2, but outcomes in Ilizarov group were slightly better than after internal stabilization.

## Background

End-stage osteoarthritis (OA) of the ankle joint, as well as post-traumatic, inflammatory, congenital and neurogenic deformities of the ankle joint result in pain and reduced mobility. Ankle arthrodesis is considered to be a well-accepted technique for end-stage ankle arthritis [1–16].

Ankle arthrodesis can be achieved with external fixators or internal stabilization with staples, screws, intramedullary nails or plates [1–3, 8–11, 13–17]. However, published opinions about the effectiveness of ankle arthrodesis with external and internal fixation vary [1–3, 8, 17]. Ankle arthrodesis with the Ilizarov apparatus can be particularly beneficial in patients with poor status of the skin and soft tissues, large or multi-planar deformities, shortening of the limb and inadequate-quality of bone. Ilizarov apparatus can be used for the distraction or compression of the joint and, if necessary, also for the correction of its axis; another advantage of this method stems from the fact that the operated limb can be bear weight early after the surgery. The drawbacks of ankle arthrodesis with the Ilizarov fixation include long wear time of the apparatus, higher cost than in the case of internal stabilization, as well as the complexity of the device and surgical technique [1, 2, 8, 9, 17]. Ankle arthrodesis with internal stabilization is feasible in patients with good quality of bones and soft tissues, minor deformities without concomitant limb shortening. Internal fixation often requires less extensive and less complex surgery than in the case of the Ilizarov apparatus. However, it is also associated with the risk of soft tissue necrosis, destabilization of ankle fusion and revision arthrodesis [2, 3, 8].

Ankle osteoarthritis interferes substantially with the activities of daily living [7, 8]. Pain and functional impairment of affected limb are principal factors that motivate patients with ankle osteoarthritis to undergo surgical treatment [8, 18]. The surgery is effective whenever it results in attenuation of pain and contributes to an improvement of the limb function [5, 7, 8, 18, 19].

An optimal method for ankle arthrodesis should be associated with minimum postoperative pain, low morbidity rate, short hospital stay and good functional outcome.

All of the published studies report the union rate, rate of complications, and the analysis of functional outcomes independently for each of the two surgical procedures - Ilizarov stabilization and internal stabilization [1, 3–11, 20, 21]. In the previous work, the authors performed a radiological evaluation of ankle arthrodesis with Ilizarov stabilization compared to internal fixation [21]. There is no work comparing rate of complications, pain level, period of hospitalization, and functional outcome in functional outcome in Foot and Ankle Ability Measure (FAAM) scale, in group with Ilizarov stabilization and internal fixation of ankle arthrodesis.

A better understanding of the impact of the type of ankle joint arthrodesis stabilization on clinical results in the form of the rate of complications, pain level, period of hospitalization, and functional outcome in FAAM scale will allow an easier decision on which type of stabilization to choose.

We assessed whether the type of stabilization after ankle arthrodesis will affect: (1) functional outcome in Foot and Ankle Ability Measure (FAAM) scale, (2) pain level, (3) period of hospitalization, (4) rate of complications.

## Methods

**Fig. 1:**
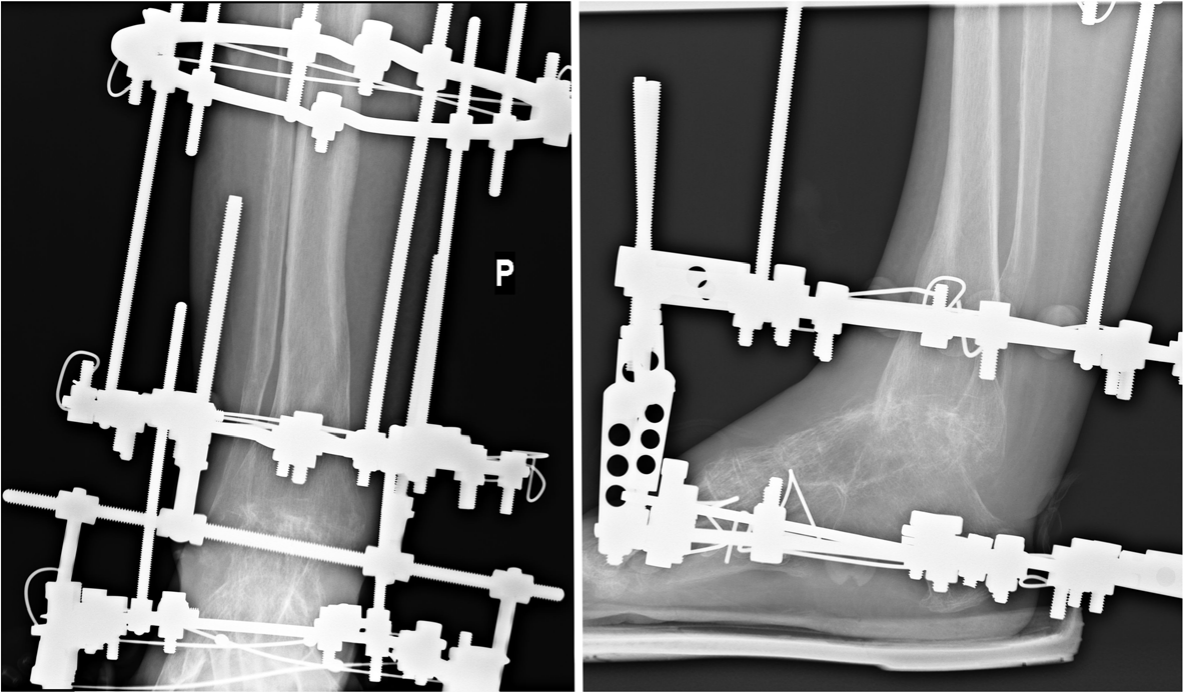
Patient with ankle joint arthrodesis with Ilizarov fixator stabilization

**Fig. 2:**
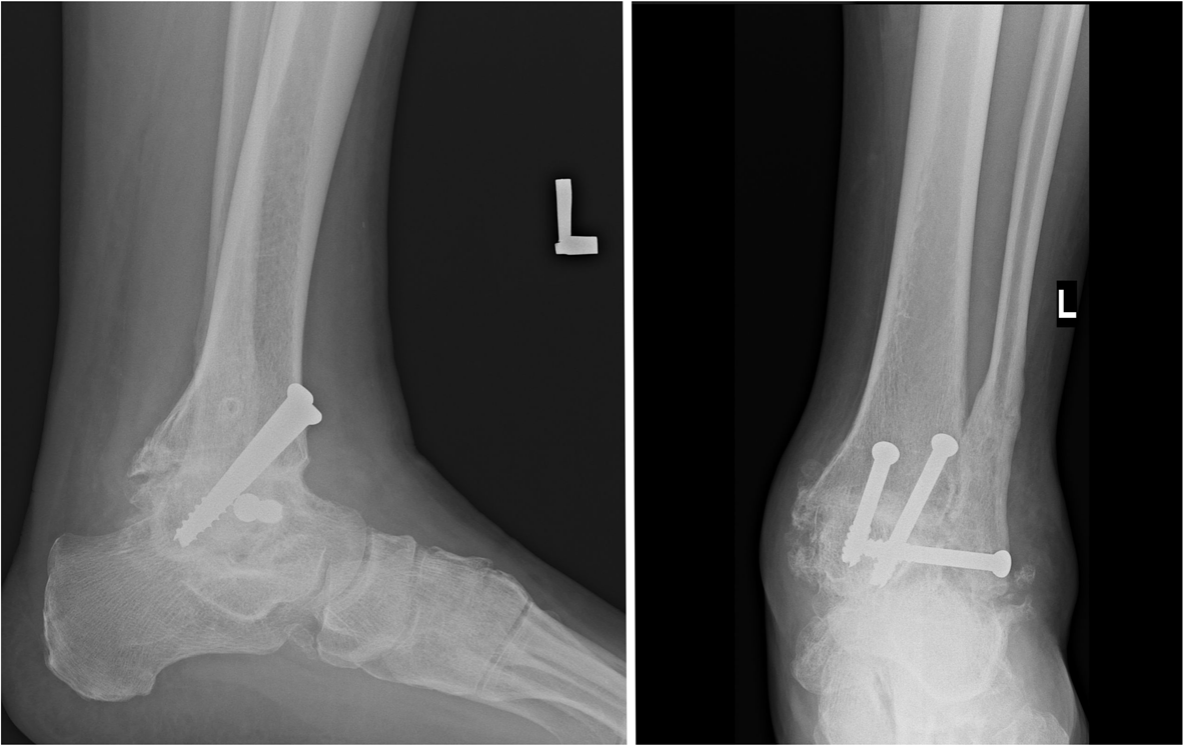
Patient after ankle joint arthrodesis with screws stabilization

We retrospectively studied all 55 individuals who had ankle arthrodesis with Ilizarov stabilization (group 1), (Fig. 1) or internal fixation with cannulated screws (group 2), (Fig. 2) at our hospital between 2007 to 2015.

Ankle arthrodesis were performed for patients with included end-stage primary or secondary (neurogenic, post-traumatic, congenital, rheumatoid) osteoarthritis of the ankle.

Ankle arthrodesis with Ilizarov fixation was always performed for: patients with bad quality of bones (infections, loss, osteoporotic), patients with bad quality of soft tissues (inflammation, necrosis, fistulas, vascular lesions, ulcers, scars, skin lesions, trophic changes), patients with multi-planar deformities or deformities > 15^0^ in one plane, patients with infection. In other patients we performed ankle joint arthrodesis with either internal stabilization or external Ilizarov stabilization. Ankle arthrodesis with internal stabilization were prefered for: patients with normal quality of bones and soft tissues, patients without concomitant disease, patients with deformities < 5^0^, and for patients well cooperated [21].

Deformities of the ankle was determined by the AP and lateral view X-ray of the ankle in weight bearing. The angle between the line corresponding to long axis of the tibia and a line corresponding to the long axis of the talus was determined in the AP projection, and the angle between a line corresponding to the long axis of the tibia and a line perpendicular to the long axis of the talus was measured in the lateral projection. Axis of the ankle t in the AP and lateral view was defined using digitalized x-rays and measurement tools [21].

Inclusion criteria consisted of the performance of the ankle arthrodesis with either an Ilizarov external fixator or internal stabilization with screws with greater than 24 months of follow up; documentation of the etiology of the ankle pathology, demographic data, rate of complications, pain level (VAS),period of hospitalization, and functional outcome in the FAAM scale. Exclusion criteria consisted of the lack of an ankle joint arthrodesis with internal fixation with screws or Ilizarov stabilization, failure to meet all inclusion criteria, Charcot neuroarthropathy, patients with multiple location of joint injuries and those with associated procedures during the surgical intervention.

Patients were enrolled into the study based on physical examination, medical history, analysis of medical records performed before and after treatment. All participants in the study knew about the voluntary nature of participation in the study. All patients gave their consent to participate in the study, complete questionnaires, and process personal data. The underage consent for the study was signed by their guardians. The study was accepted by the local Bioethics Commission.

In years 2007–2015, ankle arthrodesis were performed for 55 patients (24 with Ilizarov fixation, 31 with internal screws stabilization). In the group 1 (Ilizarov stabilization), 1 patient (4%) was not included because < 24 months follow-up, 1 patient (4%) was excluded because of bilateral ankle injuries and 1 patient (4%) was excluded because of lack of data. These 21 patients had a mean follow-up of 45 months (range, 24 to 108 months). In group 2 (internal fixation), 2 patients were not included because < 24 months follow-up (6%), 2 (6%) were excluded because of missing patient data, and 1 (3%) was not included because of bilateral ankle injuries. These 26 patients were evaluated at a mean follow up of 47 months (range, 24 to 104 months).

In all patients, the surgery was performed in a supine position, with peri-operative antibiotic administration, using a tourniquet (320 mmHg). For an ankle joint fusion we performed anterior approach. To achieve compression at the ankle joint we used Ilizarov external fixator (group 1) or cannulated screws (group 2). The construction of the Ilizarov apparatus (group 1) was as follows: foot ring fixed with 3 Kirschner wires with olives to the calcaneus and to distal part of metatarsal bones, distal ring with 2 Kirschner wires and proximal with 3 Kirschner wires. In group 2 we used 4 cannulated screws, placed in the talus and in the distal part of tibia, to achieve compression. All individuals in Ilizarov group and in group 2 were operated by 3 surgeons. Patients from Ilizarov group start walking with full possible weight bearing in day 1 after surgery. The minimum time to remove Ilizarov fixator was 9 weeks. After Ilizarov stabilization removal, patients walked in a walker-boot orthosis for a minimum of 6 weeks. After surgery, individuals from group 2 remained in a cast with non weight bearing for a minimum of 6 weeks, next increased weight bearing in a walker-boot orthosis for the next 6 weeks [21].

Clinical outcomes were measure by: (1) functional outcome in Foot and Ankle Ability Measure (FAAM) scale, (2) pain level, (3) period of hospitalization, (4) rate of complications, in Ilizarov fixator stabilization (group 1) or internal screws stabilization (group 2).

These evaluations were performed based on preoperative data and at postoperative follow-up clinic visits.

The number of complications in each patient was determined on the basis of medical documentation and history taking. Then, the mean number of complications per patient has been calculated for both study groups.

**Fig. 3:**
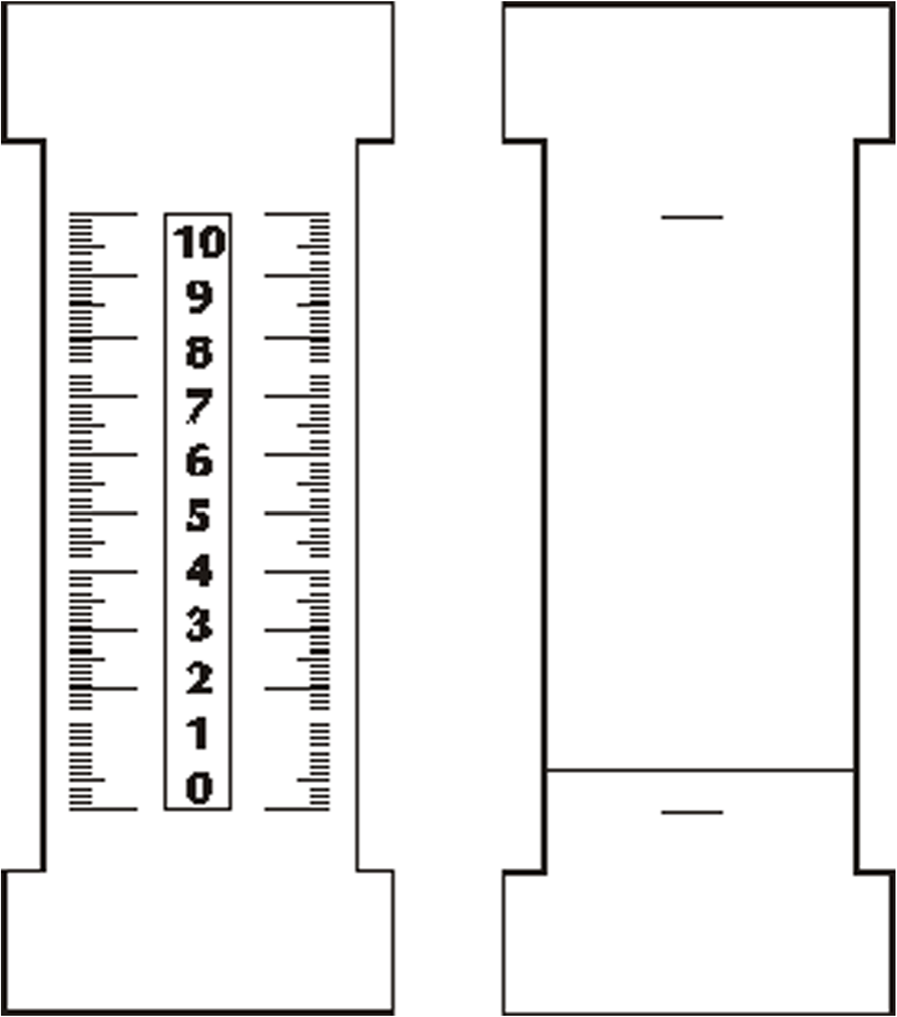
Visual analogue scale (VAS) for pain; Author’s own material

The severity of preoperative and postoperative pain was determined with visual analogue scale (VAS), from 0 to 10 (Fig. 3). Then, mean preoperative and postoperative VAS scores have been calculated. Postoperative VAS scores were measured at the final follow up.

The information on the length of hospital stay was extracted from each patient’s medical documentation. Then, mean length of preoperative and postoperative hospital stay has been calculated for both study groups.

The FAAM score included a functional assessment for daily life (21 items) [18]. Each response was graded from 0 to 4 (0 = impossible and 4 = no difficulty); if the patient did not participate in any of the activities proposed on the questionnaire for a reason other than ankle injury, this item was removed. Adding the points of the corresponding items gave the FAAM overall scores (ranged from 0 to 100) [18]. Subsequently, mean postoperative FAAM scores have been determined for both study groups, at the final follow up.

Mann–Whitney U-test, Students t-test and analysis of variance (ANOVA) were used to analyze the statistical differences between mean values of variables. For assess normal distribution the Kolmogorov–Smirnov test was performed. Statistica 10.0 software was used for all analyses with significance level of α = 0.05.

## Results

47 patients (21 in group with Ilizarov stabilization and 26 in group with internal stabilization) of both sexes (16 females, 31 males) were evaluated in the study. The average time to follow-up was 45 months (24–108) in group 1 and 47 months (24–104) in group 2. The demographic characteristic for patients in group 1 and group 2 was no statistically significant difference (Table 1). The incidence of risk factors such as smoking, overweight, and diabetes was similar in both groups of patients. We achieved ankle fusion in 100% of individuals after Ilizarov fixation and in 85% with internal stabilization [21].

**Table 1:** Patient Demographics/Characteristics

Variable	Group 1 - Ilizarov external fixator (*N* = 21)	Group 2 - internal stabilization (*N* = 26)
Age	44(17–65)	47 (17–67)
Sex	14 (66.6%) male	17 (65.4%) male
follow-up (months)	45 (24–108)	47 (24–104)
Disease diagnosis		
Primary OA	2 (9.5%)	3 (11.5%)
Secondary OA		
post-traumatic	10 (47.6%)	15 (57.7%)
rheumatoid	0 (0%)	1 (3.8%)
congenital	4 (19%)	3 (11.5%)
neuropathic	5 (23.8%)	4 (15.4%)
FAAM score [0–100]	79.38 (56–88)	70.11 (49–91)
Pain level before surgery	4.69 (0–10)	4.71 (0–10)
Pain level after surgery	1.5 (0–5) ^1^	2.9 (0.5–7) ^1^
rate of complications [number of complications/ patient]	0.62 (0–2)	0.58 (0–2)
period of hospitalization [days]	5.29 (4–8)	5.71 (4–9)

^1^statistical difference between the group (*p* < 0,05)

Total number of complications in Ilizarov group was 13, which corresponded to 0.62 complications per patient on average (range 0–2). In group 2 there were 15 complications, which corresponded to 0.58 complications per patient on average (range 0–2) (Table 1). The intergroup difference in the average rate of complications turned out to be not statistically significant (*p* = 0.066).

In group with Ilizarov external fixator stabilization infections around implants (pin site infections) appeared 8 times (61.5% of adverse events). In 6 cases the infection was controlled with local and systemic administration of antibiotics. Two patients required surgical resection of the Kirschner wire and debridement of the site of infection. Vascular injury occurred two times (15.4% of adverse events) and required repair the vessel during surgery by us (peroneal artery). Soft tissue necrosis occurred in three cases (23.1% of all adverse events). These patients were treated with wound dressings and pharmacotherapy.

In group internal stabilization with screws infections appeared 2 times (13.3% of adverse events). In 1 case the infection was controlled with local and systemic administration of antibiotics. One patient required surgical resection of sinus and debridement of the site of infection. Nonunion appeared 4 times (26.7% of adverse events). Four patients who did not achieve a fusion after the primary arthrodesis were subjected to revision with Ilizarov fixation with complete fusion in all these cases. Deformation within the ankle joint with destabilization of ankle fusion occurred 4 times (all of this patients had nonunion) (26.7% of adverse events), in all cases were corrected with the Ilizarov fixation. Soft tissue necrosis occurred 5 times (33.3% of adverse events). One patient required plastic surgery; others were treated with wound dressings and pharmacotherapy.

In Ilizarov group the mean VAS pain level before treatment was 4.69 (0–10). The mean VAS pain level after treatment was 1.5 (0–5). This difference was statistically significant (*p* = 0.037). In group 2 the mean VAS pain level before treatment was 4.71 (0–10). The mean VAS pain level after treatment was 2.9 (0.5–7), (Table 1). This difference was statistically significant (*p* = 0.044). The comparison between the average scores on the VAS pain level before treatment of the group 1 and group 2 revealed no statistically significant differences. After treatment group with internal stabilization had significant higher VAS pain level (Table 1), (*p* = 0.044).

In group 1 the mean time of hospitalization was 5.29 (4–8) days. In group 2 the mean time of hospitalization was 5.71 (4–9) days. This difference was not statistically significant (Table 1), (*p* = 0.517).

In Ilizarov group the mean functional outcome in FAAM scale was 79.38 (56–88). In group 2 the mean functional outcome in FAAM scale was 70.11 (49–91). FAAM functional outcome was higher in the group 1 but not statistically over the group 2 (Table 1), (*p* = 0.458).

## Discussion

Ankle arthrodesis with the Ilizarov apparatus can be particularly beneficial in patients with poor status of the skin and soft tissues, large or multi-planar deformities, shortening of the limb and inadequate-quality of bone, it also allows to union achieve in a larger percentage of cases [9–11, 21]. Ankle joint arthrodesis with internal fixation is feasible in patients with good quality of bones and soft tissues, minor deformities without concomitant limb shortening.

Choice of an appropriate method for ankle joint arthrodesis, providing maximal attenuation of postoperative pain, minimal morbidity, short hospital stay and good functional outcome, is a key component of treatment planning.

In this study we analyzed whether the type of arthrodesis fixation will affect (1) functional outcome in Foot and Ankle Ability Measure (FAAM) scale, (2) pain level, (3) period of hospitalization, (4) rate of complications. To the best of our knowledge, none of the previous studies analyzed the clinical outcomes of ankle arthrodesis according to the fixation method.

In the previous study, the authors performed a radiological evaluation of ankle arthrodesis with Ilizarov fixation compared to internal fixation [21]. This study demonstrated that Ilizarov stabilization of ankle arthrodesis is associated with lower ankle joint misalignment and lower prevalence of adjacent-joint OA, and with higher fusion rates (100%) than after internal fixation with screws(88%) [21].

In the available literature there is a large discrepancy in the number of complications per patient after ankle arthrodesis [1, 5, 9, 11, 22]. In the study conducted by Katsenis et al., including 21 patients subjected to Ilizarov treatment, 41 minor (treated conservatively) and 20 major complications (treated surgically) occurred. Another seven complications, requiring four additional operations, were noted after the removal of the circular frame [9]. Altogether, this corresponded to an average number of 3.24 complications per patient. In another study of 22 patients subjected to ankle joint arthrodesis with Ilizarov fixation, complications occurred in 11 cases; this included two nonunions that healed after revision and application of a renewed frame, and four pin track infections [11]. Consequently, mean number of complications documented in this study amounted to 0.5 per patient. Fragomen examined 91 patients after ankle arthrodesis with Ilizarov fixation; the list of postoperative complications included nonunions (*n* = 15), tibial stress fractures (*n* = 6), malunions (*n* = 4), broken fixations (*n* = 3), cellulitis (*n* = 3), neural injuries (*n* = 2), and isolated cases of arterial embolus, severe deep infection, knee flexion contracture and calcaneal collapse [1]. In the study conducted by Chahal et al., 5.7% of patients developed infection after internal fixation procedure [5]. SooHoo et al. reported complications in 11% of patients after ankle arthrodesis [22]. Our study groups did not differ significantly in terms of the average number of complications per patient (in group 1–0.63; in group 2–0.59), and the morbidity rates were similar or smaller, than those reported previously (0.14–3.24) [1, 5, 9, 11, 22]. This shows that our treatment results in terms of the number of complications are similar or slightly better than the results presented in the quoted articles. The number of complications is similar regardless of the stabilization methods of ankle arthrodesis.

Most common complication in external fixator treatment are infections around implants [8, 11]. Also in our study, in group with Ilizarov external fixator stabilization infections around implants was the most common complication (61.5% of adverse events). Most common pathogen in pin-site infection is *Staphylococcus aureus* [8]. Internal or external stabilization loosening may result from infection or long-term impact of micromitions [8]. External fixator has greater rigidity which results in better biomechanical properties of ankle fusion than those observed after an internal fixation [23]. These were better biomechanical properties and greater rigidity of the Ilizarov fixator which likely contributed to the lack of nonunions in our group of patients subjected to external fixation.

None of the previous studies analyzed the effect of fixation type of ankle arthrodesis on the VAS pain level before and after surgery. According to some researchers, nonunion after ankle joint arthrodesis may be associated with pain [5, 19]. In the study conducted by Dalat et al., mean pain scores of patients subjected to ankle arthroplasty and those after ankle arthrodesis were 16.6 and 24.3 out of 100, respectively [7]. We did not find significant intergroup differences in preoperative pain scores. After the surgery, however, the pain scores of patients subjected to Ilizarov fixation decreased more, than the VAS pain scores of those after internal fixation. Patients in the group with screws stabilization after the surgery were taking more analgesics. The primary aim of this surgery is sound bony fusion. If it happened most of the preoperative pain will disappear. In the internal fixation group there were 4 cases complicated by nonunion. Ilizarov fixation might reflect lesser impact of this method on musculoskeletal system than in the case of ankle arthrodesis with screws. Also the possibility of full loading of the operated limb in day 1 after Ilizarov surgery can better influence the biomechanics of the lower limbs compared to internal stabilization. A long period of non-weight bearing reduces muscle strength and limits joint mobility, which can cause more pain.

Our study groups did not differ significantly in terms of the length of hospital stay. This might be associated with similar morbidity rates after the two types of fixation and with the fact that subjects from both groups at the same time after surgery were taught to walk on crutches. None of the previous studies analyzed the effect of fixation type of ankle arthrodesis on the period of hospitalization.

There was no statistically significant difference in FAAM scores for patients in Ilizarov group and group 2. In the group evaluated by Dalat et al., mean postoperative FAAM scores of patients with ankle joint endoprosthesis and ankle arthrodesis were 77.6 and 63.4 points, respectively [7]. Mean FAAM score for a group of 164 patients with various pathologies of the feet and ankle examined by Martin et al. was 74.9 points [18]. In Houdek’s study, mean FAAM score of 31 patients subjected to bilateral ankle fusion with internal fixation was 70 points [13]. Strasser and Turner documented the average FAAM score of 81.5 points for a group of 30 individuals after ankle arthrodesis with external fixation [14]. According to Hendrickx et al., mean FAAM score of 66 subjects subjected to ankle fusion with internal fixation was 69 points [15]. Katsenis et al. followed-up 21 patients after ankle arthrodesis with Ilizarov fixation; excellent functional outcomes were documented in 15 patients, good in three, fair in two and poor in only one [9]. In our research, patients with Ilizarov stabilization presented similar functional FAAM scores, compared to internal fixation group. FAAM scores of our individuals were slightly higher than those reported by other researchers. This shows that our treatment results in terms of FAAM scores are similar or slightly better than the results presented in the quoted articles. In the available literature, the results on the FAAM scale after ankle arthrodesis are similar regardless of the stabilization methods and are comparable to FAAM scores after ankle joint endoprosthesis. According to some researchers, functional outcomes of ankle joint arthrodesis are not good [5, 19]. In the study performed by Chahal et al., patients after ankle arthrodesis with internal fixation had functional scores below the values of average American population [5]. The same study demonstrated that patients with non-union presented with worse functional outcomes [5]. Also in another study, patients with non-union after ankle arthrodesis experienced dysfunction more often than those with normal healing [19 From the literature review and our results it can be concluded, that patients after ankle arthrodesis can have worse functional outcomes than the general population. This may be due to the fact that patients after ankle arthrodesis have higher pain sensations and greater disability compared to the general population.

Some surgeons, in the case of degenerative changes of ankle and subtalar joints prefers hindfoot arthrodesis[24–26]. A tibiotalocalcaneal arthrodesis allows for fusion of two joints during one operation, which is beneficial in patients with changes of ankle and subtalar joints. However, in the case of this procedure, the biomechanics of the lower limb are more disturbed than after the ankle joint arthrodesis. A wide range of tibiotalocalcaneal arthrodesis techniques had been described [24–26]. In case of tibiotalocalcaneal arthrodesis with open surgical techniqes there is a big risk of complications like poor wound healing and infections [24–26]. The arthroscopic approach for tibiotalocalcaneal arthrodesis gives good results with a small rate of complications [24–26].

One of the limitations of our work is the lack of performance of CT for the evaluation of ankle union. Normally, we estimated union, based on X-ray examination and clinical examination. We do not routinely performed CT, because of the high radiation dose, the poor quality of the CT image with metal artifacts and the long waiting period for the study. One of the weaknesses of our work is comparing two groups of patients with different intensity of pathology within the ankle joint. It is difficult to collect quite numerous groups of patients with different methods of stabilization after ankle arthrodesis, which is why in our work we evaluated all patients with stabilization with the Ilizarov apparatus. Some of the patients treated with the Ilizarov method had a poor quality of bone and soft tissues, and had the initial deformity of the ankle joint. However, some patients treated with the Ilizarov method did not have poor quality of bones or soft tissues, and the ankle joint was not deformed. Also, the difference (24–108 month) in the time to follow up within the groups can bring insecurity to the study and is a weakness. Surgery for all of the patients performed 3 surgeons. The strong points of this study are the same surgery protocol, homogeneity of rehabilitation protocol in both groups, and the fact that there are no studies comparing functional results, rate of complications, pain level, and period of hospitalization, after ankle joint arthrodesis with Ilizarov stabilization and internal fixation.

In this work, we compared only clinical results after ankle joint arthrodesis with Ilizarov stabilization and internal fixation, as in other works prepared by us for publication, we evaluated radiological outcomes [21], sports activity and pedobarographic evaluation of gait and posture in patients after ankle arthrodesis with Ilizarov stabilization and internal stabilization.

The goal of ours study was to provide the surgeon and the patient with a realistic perspective on treatment outcome in relation to clinical comparison of ankle arthrodesis with external and internal stabilization.

## Conclusions

VAS pain level after Ilizarov external stabilization of ankle joint arthrodesis was lower than after internal fixation. Period of hospitalization, FAAM functional score and rate of complications were not statistically significant between both groups.

## Abbreviations

FAAM scale: Foot and Ankle Ability Measure scale
